# Genetic and environmental variance of renal parenchymal thickness: a twin study

**DOI:** 10.3325/cmj.2013.54.550

**Published:** 2013-12

**Authors:** David Laszlo Tarnoki, Adam Domonkos Tarnoki, Levente Littvay, Pal Bata, Viktor Berczi, Zsolt Garami, Kinga Karlinger

**Affiliations:** 1 Department of Radiology and Oncotherapy, Semmelweis University, Budapest, Hungary; 2 Central European University, Budapest, Hungary; 3 Houston Methodist DeBakey Heart and Vascular Center, The Houston Methodist Hospital, Houston, TX, USA; * The first two authors contributed equally to the study.

## Abstract

**Aim:**

To estimate heritability and environmental effects on renal parenchymal thickness.

**Methods:**

In this twin study, renal parenchymal thickness of 98 Hungarian healthy adult twin pairs (68 monozygotic, 30 dizygotic) without kidney disease was measured bilaterally using renal ultrasound with Esaote MyLab 70X ultrasound machine with low-frequency curved transducers (1-8 MHz).

**Results:**

In both monozygotic and dizygotic group there were more women (76.5%). Mean right and left renal parenchymal thickness was 1.32ƒ?%¶+ƒ?%0.33 cm and 1.62ƒ?%¶+ƒ?%0.31 cm, respectively. Age- and sex-adjusted heritability of renal parenchymal thickness was 0.0% (95% confidence interval, 0.0 to 50.2%), shared and unshared environmental factor was 30.2% (4.1 to 55.9%) and 69.8% (45.8 to 89.5%), respectively.

**Conclusion:**

This study shows a negligible role of heritability and an important role of environmental effects in developing renal parenchymal thickness, emphasizing the importance of lifestyle for primary prevention.

Decrease in renal parenchymal thickness (RPT) is an important sign of renal disease, which is accompanied by decreased renal cortical thickness (RCT). Several renal conditions are associated with a decrease in RPT and RCT, such as chronic kidney disease, renal transplantation, or nephropathia epidemica (1-3). Parenchymal thickness indicates chronic nature of renal failure and is regarded as a more exact sonographic parameter in end-stage renal failure than renal size (4,5). There is increasing evidence that RPT is a sensible marker of acute kidney damage (6). Ultrasound assessment of RPT can reliably differentiate between patients with acute or chronic renal failure (7). RCT and RPT are regarded as early and sensitive morphological markers for the early diagnosis of atherosclerotic kidney disease and correlate with the coronal diameter, renal volume, intraparenchymal resistance indices, glomerular filtration rate, and proteinuria (8).

Although the change in RPT has been comprehensively studied in acute and chronic renal diseases, it has not been studied extensively in healthy people. There is little knowledge on whether the normal RPT, an index for studying the health status of the kidney, is only influenced by environmental factors or if it is also influenced by genetic factors. We hypothesized that the previously described normal age-related change in RPT can be also genetically influenced (9).

## Methods

### Participants and study design

This cross-sectional twin study included 196 healthy adult twins (68 monozygotic, 30 dizygotic twin pairs) recruited from the Hungarian Twin Registry in 2009 and 2010 (10). We considered only the same-sex dizygotic twin pairs to avoid bias of the heritability estimates in the presence of sex specific or X chromosome effects. Exclusion criteria included history of acute or chronic renal disease, pregnancy, and any foreseeable lack of compliance with test procedures. None of monozygotic and dizygotic twins met these criteria, therefore all considered pairs (nƒ?%=ƒ?%98) were included in the study. Instead of genotyping for zygosity classification, we used a multiple-choice self-reported seven-part questionnaire with >99% accuracy (11). All participants gave informed consent. The study was approved by the Ethical Committee of Semmelweis University and was conducted in full compliance with regulations of the Declaration of Helsinki.

### Renal ultrasound assessment

Renal ultrasound testing was conducted at the Department of Radiology and Oncotherapy, Semmelweis University in 2009 and 2010. Participants completed a questionnaire in order to identify clinical symptoms and to obtain complete medical history.

Limited renal sonography was performed using B-mode ultrasonography (Esaote MyLab 70X Vision, Esaote, Genova, Italy) equipped with a curved array transducer (1-8 MHz, CA431). The gray-scale amplification gain, the time-gain compensation curve, and focus number (adjusted at the level of the kidney) were adjusted to acquire the best images of the kidneys. The examinations were performed by the same experienced sonographer. All participants were well hydrated and had full bladders at the time of the examination. Renal measurements were performed with patients in the supine position or in the contralateral decubitus position according to standard guidelines (12). Sagittal plane images were obtained either from the long-axis view, using a subcostal approach with the patient in the supine position, or from the contralateral decubitus position view, using a posterior approach with the patient in the contralateral decubitus position. All kidneys were completely visible and measurable. Parenchymal thickness was defined as the distance between the cortex-perirenal fat interface (capsule) and the sinus-pyramidal apex interface, and was measured at the middle third portion of the kidney. Standardized static original digital images of both kidneys were recorded and RPT measurements were obtained prospectively using electronic calipers at the time of scanning. These images were retrospectively examined by a specialized radiologist blinded to the participantsƒ?(tm) twinship and clinical characteristics in order to confirm the accuracy of the RPT measurements.

### Statistical analysis

Descriptive analysis (mean, standard deviation, and percentage for categorical variables) was conducted by SPSS Statistics 17 (SPSS Inc., Chicago, IL, USA). *P* values lower than 0.05 were considered significant. A descriptive estimate of the genetic influence on a single trait and of the genetic correlation between different traits in monozygotic and dizygotic pairs was calculated using the within-pair co-twin correlations. Greater levels of monozygotic than dizygotic within-pair similarity indicated a genetic influence on a phenotype, while similar co-twin correlation of dizygotic and monozygotic twins indicated an environmental influence. Deviations from perfect monozygotic co-twin correlations were attributed to the unshared environment. Structural equation modeling was performed using the Mplus Version 6.1 (MuthÆ(c)n & MuthÆ(c)n, Los Angeles, CA, USA). Weighted least squares estimation was used due to the categorical nature of dependent variable (13). Empirical confidence intervals were calculated with a Bollen-Stine Bootstrap (14). Univariate quantitative genetic modeling was performed to calculate the percentage of phenotypic variance of heritability (A), shared (C), and unshared (E) environmental effects (univariate ACE analysis). The additive genetic component (A) measures the effect of genes at multiple loci or multiple alleles at one locus. The shared environmental component (C) estimates the contribution of the common family environment for both twins (eg, familiar socialization), whereas the unshared environmental component (E) estimates the effects that separately influence each individual twin and also accounts for measurement errors. The ACE model makes realistic assumptions to estimate these components (15).

## Results

### Descriptive analysis of the twin cohort

There were more women in both groups of twins (76.5%). Dizygotic twins were significantly older than monozygotic twins (*P*ƒ?%<ƒ?%0.05), therefore all analyses were age corrected. Mean right and left RPT showed low correlation (rƒ?%=ƒ?%0.209, *P*ƒ?%<ƒ?%0.01). No significant difference was observed between monozygotic and dizygotic twins in other clinical and ultrasonographic characteristics (Table 1).

**Table 1:** Clinical and ultrasonographic characteristics according to zygosity

	Total	Zygosity	
		monozygotic	dizygotic
Participants, n	196	136	60
Women:men ratio	150:46	104:32	46:14
Age, years (meanƒ?%¶+ƒ?%standard deviation)	44.2ƒ?%¶+ƒ?%16.7	42.3ƒ?%¶+ƒ?%16.9ƒ?	48.5ƒ?%¶+ƒ?%15.5ƒ?
Hypertension*, n (%)	62 (31.6)	44 (32.4)	18 (30.0)
Diabetes^ƒ?ó^, n (%)	10 (5.1)	9 (6.6)	1 (1.7)
Hyperlipidemia^¶õ^, n (%)	44 (22.4)	27 (19.9)	17 (28.3)
Body mass index, kg/m^2^ (meanƒ?%¶+ƒ?%standard deviation)	25.7ƒ?%¶+ƒ?%4.9	25.6ƒ?%¶+ƒ?%5.0	25.9ƒ?%¶+ƒ?%4.7
Current smokers, n (%)	27 (13.8)	19 (14.0)	8 (13.3)
Stroke, n	4 (2.0)	4 (2.9)	0 (0.0)
Myocardial infarction, n (%)	7 (3.6)	5 (3.7)	2 (3.3)
Mean right renal parenchymal thickness, cm (meanƒ?%¶+ƒ?%standard deviation)	1.32ƒ?%¶+ƒ?%0.33	1.32ƒ?%¶+ƒ?%0.32	1.33ƒ?%¶+ƒ?%0.33
Mean left renal parenchymal thickness, cm (meanƒ?%¶+ƒ?%standard deviation)	1.62ƒ?%¶+ƒ?%0.31	1.60ƒ?%¶+ƒ?%0.33	1.67ƒ?%¶+ƒ?%0.30

*Defined according to the clinical history and patients on anti-hypertensive medication.

ƒ? *P*ƒ?%<ƒ?%0.05 vs dizygotic.

ƒ?óDefined according to the clinical history and current definition, two fasting glucose measurements above 126 mg/dL (7.0 mmol/L).

¶õDefined as elevated concentrations of any or all of the lipids in the plasma, according to the clinical history.

### Heritability analysis of RPT in twins

The possible role of zygosity in the prevalence of RPT was estimated by age- and sex-adjusted ACE analysis. Age and sex-adjusted heritability of right RPT was 38.5% (95% confidence interval [CI], 1 to 68.4%), shared environmental effects were 0.0% (95% CI, 0.0 to 45.3%) and unshared environmental effects were 61.5% (95% CI, 35.5 to 86.6%). Left RPT showed no additive genetic effects ƒ?" 0.0% (95% CI, 0.0 to 10.2%), and shared and unshared environmental effects were 8.2% (95% CI, 0.0 to 32.7%) and 91.8% (95% CI, 6.9 to 100%), respectively. The difference in variations between left and right RPT was non-significant (*P*ƒ?%>ƒ?%0.05), and the heritability of right RPT was marginally significant (lower value of 95% CI was almost 0).

**Figure 1:**
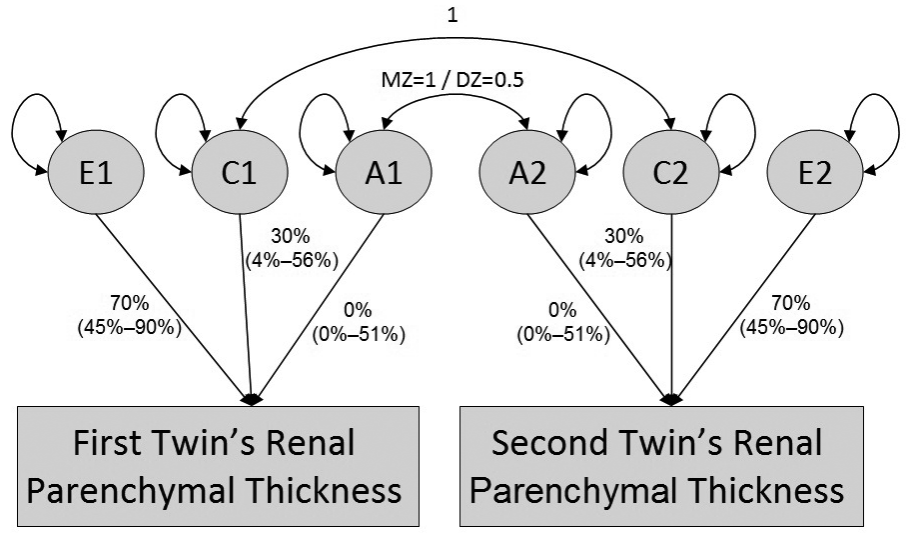
Univariate additive genetic (A), common environmental (C), and unique environmental (E) influences analysis of renal parenchymal thickness (RPT). Rectangles denote the observed variables (right and left RPT) and circles denote the latent variables of A, C and E. Curved arrows denote correlations (fixed at the highlighted values). Straight arrows signify the estimated impact of the latent factor on the variance of the observed phenotype.

In conclusion, genetic factors did not contribute to the variance of RPT and the largest proportion of total variance was attributable to unshared environmental factors (Figure 1).

## Discussion

Our analysis did not support the hypothesis of heritability of RPT, since the greatest part of the variance was explained by unshared environmental components. To the best of our knowledge, this is the first twin study investigating the relative contribution of genetic and environmental factors to RPT. Genetic studies using the twin design are based upon the assumption that twins are representative of the general population for the outcomes being studied.

The normal kidney length is around 110ƒ?%¶+ƒ?%20 mm, with great interindividual differences (16). Several studies have used pathologic material to describe the variation of kidney size relative to sex, age, and ethnicity (17,18). We took these factors into account by adjusting the univariate models by age and sex. The study sample consisted of only Caucasian participants.

Reduced kidney size is partly related to renovascular diseases, and correlates with the evolving nature of kidney disease (19). We also observed a slight difference between right and left RPT. The left RPT was greater (eg, hypertrophied column of Bertin, persistence of fetal lobation, and dromedary hump, which are usually present at the middle third portion of the left kidney), and the greatest RPT was measured on that side. This interrenal difference has been previously shown (8,9,20).

A number of reports have shown that the amount of renal parenchyma decreases by about 10% per decade of advancing age for both kidneys, with the highest decrease rate in the sixth and the seventh decades independent of sex (8,9). Loss of renal parenchyma in the elderly is compensated for by an increase in peripelvic fat (9,21,22). Our study provided evidence that genetic factors did not substantially contribute to this phenomenon, however, diverse environmental factors (eg, lifestyle: cigarette smoking, nutrition, lack of physical exercises) had a tremendous influence (70%). Our study is in accordance with previous findings that parenchymal thickness was a good marker of atherosclerotic renal disease, considering that atherosclerotic phenotypes were also mainly influenced by environmental factors (23-27). On the basis of such results, we believe that RPT should be monitored as an important risk factor in individuals with an unhealthy lifestyle.

Our study has several limitations. RPT was visualized and measured by ultrasonography, rather than by computed tomography or magnetic resonance imaging. Ultrasonography is a relatively inexpensive imaging method that has been successfully used to screen large populations for the presence of kidney disease (28). It is readily available, uses no ionizing radiation, can be performed bedside, used repeatedly, and is accurate in determining kidney measurements (29). It has a weakness of inter-observer variability but in this study all participants were evaluated by the same sonographer and the results were later checked by a professional radiologist. Additional limitations include the relatively small number of participating dizygotic twins compared to usual twin studies, which may lead to statistical errors in the ACE analysis by increasing the E variance.

In conclusion, our study suggests that heritability has a negligible role for the development of RPT in a healthy twin population, while shared and mainly unshared environmental effects respectively accounted for 30% and 70% of the studied variations. The findings support the view that environmental effects may be a primary cause of RPT changes, underscoring the importance of lifestyle in primary prevention.

## References

[1] Leonard MB (2007). A structural approach to the assessment of fracture risk in children and adolescents with chronic kidney disease.. Pediatr Nephrol.

[2] Mussa A, Porta F, Gianoglio B, Gaido M, Nicolosi MG, De Terlizzi F (2007). Bone alterations in children and young adults with renal transplant assessed by phalangeal quantitative ultrasound.. Am J Kidney Dis.

[3] Paakkala A, Kallio T, Huhtala H, Apuli P, Paakkala T, Pasternack A (2002). Renal ultrasound findings and their clinical associations in nephropathia epidemica. Analysis of quantitative parameters.. Acta Radiol.

[4] Roger SD, Beale AM, Cattell WR, Webb JA (1994). What is the value of measuring renal parenchymal thickness before renal biopsy?. Clin Radiol.

[5] Mazzotta L, Sarteschi LM, Carlini A, Antonelli A (2002). Comparison of renal ultrasonographic and functional biometry in healthy patients and in patients with chronic renal failure.. Arch Ital Urol Androl.

[6] Deng GY, Sun JJ, Wang P, Mo JC (2010). Renal parenchymal thickness and urinary protein levels in patients with ureteropelvic junction obstruction after nephrostomy placement.. Int J Urol.

[7] Ozmen CA, Akin D, Bilek SU, Bayrak AH, Senturk S, Nazaroglu H (2010). Ultrasound as a diagnostic tool to differentiate acute from chronic renal failure.. Clin Nephrol.

[8] Mounier-Vehier C, Lions C, Devos P, Jaboureck O, Willoteaux S, Carre A (2002). Cortical thickness: an early morphological marker of atherosclerotic renal disease.. Kidney Int.

[9] Gourtsoyiannis N, Prassopoulos P, Cavouras D, Pantelidis N (1990). The thickness of the renal parenchyma decreases with age: a CT study of 360 patients.. AJR Am J Roentgenol.

[10] Littvay L, Metneki J, Tarnoki AD, Tarnoki DL (2013). The Hungarian Twin Registry.. Twin Res Hum Genet.

[11] Heath AC, Nyholt DR, Neuman R, Madden PA, Bucholz KK, Todd RD (2003). Zygosity diagnosis in the a bsence of genotypic data: an approach using latent class analysis.. Twin Res.

[12] Kadioglu A (2010). Renal measurements, including length, parenchymal thickness, and medullary pyramid thickness, in healthy children: what are the normative ultrasound values?. AJR Am J Roentgenol.

[13] Muthen LK, Muthen BO. Mplus user's guide. 6th ed. Los Angeles: Muthen and Muthen; 1998-2010.

[14] Bollen KA, Stine RA (1992). Bootstrapping goodness-of-fit measures in structural equation models.. Sociol Methods Res.

[15] Littvay L (2012). Do heritability estimates of political phenotypes suffer from an equal environment assumption violation? Evidence from an empirical study.. Twin Res Hum Genet.

[16] Emamian SA, Nielson MB, Pederson JF, Ytte L (1993). Kidney dimensions at sonography: correlation with age, sex and habitus in 665 adult volunteers.. AJR Am J Roentgenol.

[17] Kasiske BL, Umen AJ (1986). The influence of age, sex, race, and body habitus on kidney weight in humans.. Arch Pathol Lab Med.

[18] Tauchi H, Tsuboi K, Okutomi J (1971). Age changes in the human kidney of the different races.. Gerontologia.

[19] Caps MT, Zierler RE, Polissar NL, Bergelin RO, Beach KW, Cantwell-Gab K (1998). Risk of atrophy in kidneys with atherosclerotic renal artery stenosis.. Kidney Int.

[20] Klare B, Geiselhardt B, Wesch H, SchÆÏrer K, Immich H, Willich E (1980). Radiological kidney size in childhood.. Pediatr Radiol.

[21] Simon Al (1960). Normal renal size: an absolute criterion.. AJR Am J Roentgenol.

[22] Lonergan ET (1988). Aging and the kidney: adjusting treatment to physiologic change.. Geriatrics.

[23] Tarnoki AD, Tarnoki DL, Stazi MA, Medda E, Cotichini R, Lucatelli P (2011). Twins lead to the prevention of atherosclerosis. Preliminary findings of International twin study 2009.. J Vasc Ultras..

[24] Tarnoki AD, Tarnoki DL, Stazi MA, Medda E, Cotichini R, Nistic‘? L (2012). Heritability of central blood pressure and arterial stiffness: a twin study.. J Hypertens.

[25] Franklin SS, Lopez VA, Wong ND, Mitchell GF, Larson MG, Vasan RS (2009). Single versus combined blood pressure components and risk for cardiovascular disease: the Framingham Heart Study.. Circulation.

[26] Jermendy G, HorvÆóth T, Littvay L, Steinbach R, Jermendy AL, TÆórnoki AD (2011). Effect of genetic and environmental influences on cardiometabolic risk factors: a twin study.. Cardiovasc Diabetol.

[27] Jermendy G, Littvay L, Steinbach R, Jermendy A, TÆórnoki A, TÆórnoki D (2011). Heritability of the risk factors characteristic for the metabolic syndrome: a twin study.. Orv Hetil.

[28] Adibi A, Emami Naini A, Salehi H, Matinpour M (2008). Renal cortical thickness in adults with normal renal function measured by ultrasonography.. Iran J Radiol.

[29] Sargent MA, Gupta SC (1993). Sonographic measurement of relative renal volume in children: comparison with scintigraphic determination of relative renal function.. AJR Am J Roentgenol.

